# Recurrence Resonance” in Three-Neuron Motifs

**DOI:** 10.3389/fncom.2019.00064

**Published:** 2019-09-11

**Authors:** Patrick Krauss, Karin Prebeck, Achim Schilling, Claus Metzner

**Affiliations:** ^1^Cognitive Computational Neuroscience Group at the Chair of English Philology and Linguistics, Department of English and American Studies, Friedrich-Alexander University Erlangen-Nürnberg, Erlangen, Germany; ^2^Neuroscience Lab, Experimental Otolaryngology, University Hospital Erlangen, Erlangen, Germany; ^3^Biophysics Group, Department of Physics, Friedrich-Alexander University Erlangen-Nürnberg, Erlangen, Germany

**Keywords:** stochastic resonance, coherence resonance, recurrent neural networks, entropy, mutual information, motifs, noise

## Abstract

Stochastic Resonance (SR) and Coherence Resonance (CR) are non-linear phenomena, in which an optimal amount of noise maximizes an objective function, such as the sensitivity for weak signals in SR, or the coherence of stochastic oscillations in CR. Here, we demonstrate a related phenomenon, which we call “Recurrence Resonance” (RR): noise can also improve the information flux in recurrent neural networks. In particular, we show for the case of three-neuron motifs with ternary connection strengths that the mutual information between successive network states can be maximized by adding a suitable amount of noise to the neuron inputs. This striking result suggests that noise in the brain may not be a problem that needs to be suppressed, but indeed a resource that is dynamically regulated in order to optimize information processing.

## 1. Introduction

Recurrent neural networks (RNN) with apparently random connections occur ubiquitously in the brain (Middleton and Strick, 2000; Song et al., 2005). They can be viewed as complex non-linear systems, capable of ongoing activity even in the absence of driving inputs, and they show rich dynamics, including oscillatory, chaotic, and stationary fixed point behavior (Krauss et al., 2019). Recently RNNs gain popularity in bio-inspired approaches of neural information processing, such as reservoir computing (Schrauwen et al., 2007; Verstraeten et al., 2007; Lukoševičius and Jaeger, 2009). Due to the built-in feed-back loops that distinguishes RNNs from networks with a pure feed-forward structure, input information that enters a RNN at some point in time can “circulate” within the network for extended periods. Moreover, a typical RNN will not simply conserve the input information in its original form, but transform it to new and possibly more useful representations at each time step. This ability of RNNs to dynamically store and continuously re-code information, as well as the possibility to combine the circulating information with new inputs, is essential for the processing of sequential data (Skowronski and Harris, 2007).

From an engineering point of view, a system that stores information by continuous re-coding has to meet two requirements: (1) The number of different codes that can be represented in the system should be as large as possible, in order to enhance the probability that some of these codes are actually useful for some read-out units and for further information processing. (2) The transformations from one code to the next should be as reproducible as possible, since otherwise information gets lost. Taken together, these two requirements are equivalent to maximizing the mutual information (MI) between subsequent states of the system.

While the transitions between subsequent network states can be made perfectly reproducible in an artificial network of deterministic neurons, it is unclear how this reproducibility is achieved in the brain, where neural computations are subject to a large degree of internal and external noise (Faisal et al., 2008; Rolls and Deco, 2010).

We therefore investigate in this work how the MI between subsequent network states depends on the level of noise added to the inputs of each neuron. For this purpose, we use one of the simplest examples of RNNs, namely the class of probabilistic three-neuron motifs with ternary connection strengths (–1, 0, 1) (Krauss et al., 2019). Strikingly, we find that the MI is not in general decreasing monotonically with the noise level, but has for certain types of motifs a peak at some optimal level of added noise.

This behavior resembles the phenomenon of Stochastic Resonance (SR), in which adding noise to the input of a sensor can enable this sensor to detect weak signals that would otherwise remain below the detection threshold (Benzi et al., 1981; Wiesenfeld and Moss, 1995; Gammaitoni et al., 1998; Moss et al., 2004; Krauss et al., 2017). Another similar effect is the so-called Coherence Resonance (CR), where the addition of a certain amount of noise to an excitable system with oscillatory response can enhance the degree of coherence in the system output (Pikovsky and Kurths, 1997; Lee et al., 1998). While the novel effect presented in this study shares with SR and CR the resonance-like dependence of some objective-function on the noise level, we consider here a free-running recurrent neural network without input and output, and without any threshold. For this reason, we will use the term “Recurrence Resonance” (RR) for this new type of effect.

In previous studies we argued that SR might be a major processing principle of the auditory system (Krauss et al., 2016; Gollnast et al., 2017) and the cerebral cortex (Krauss et al., 2018). Based on the here presented new results, we speculate that the brain may use noise more generally to optimize information processing in its different types of recurrent neural networks.

## 2. Methods

### 2.1. Probabilistic Three-Neuron Motifs

Our study is based on Boltzmann neurons (Hinton and Sejnowski, 1983) without bias. The **total input** *z*_*i*_(*t*) of neuron *i* at time step *t* is calculated as:

(1) $$\begin{array}{r} z_{i}\left(\text{t}\right)=\overset{N}{\underset{j=1}{\sum }}w_{ij\;}s_{j}\left(t\right) \end{array}$$

where *s*_*j*_(*t*) ∈ {0, 1} is the binary state of neuron *j* at present time *t*, *w*_*ij*_ is the **connection weight** from neuron *j* to neuron *i*, and *N* is the total number of neurons in the network.

In Boltzmann neurons the binary state in the next time step, *s*_*i*_(*t* + 1), is chosen randomly, with an **on-state probability**

(2) $$\begin{array}{r} p_{i}\left(s_{i}\left(t+1\right)=1\right)=\sigma \left(z_{i}\left(t\right)\right) \end{array}$$

that depends on the total input *z*_*i*_(*t*) according to a logistic function:

(3) $$\begin{array}{r} \sigma \left(z\right)=\frac{1}{1+e^{-z}}. \end{array}$$

We restrict our investigation to network motifs of three Boltzmann neurons, using discrete, ternary connection weights *w*_*ij*_ ∈ {−1, 0, 1}, where self connections *w*_*ii*_ are permitted.

In the following, we denote the **present global state** (at time *t*) of a three-neuron motif by the binary vector

(4) $$\begin{array}{r} \vec{x}=\left(s_{1}\left(t\right),s_{2}\left(t\right),s_{3}\left(t\right)\right) \end{array}$$

and its **next global state** (at time *t* + 1) by

(5) $$\begin{array}{r} \vec{y}=\left(s_{1}\left(t+1\right),s_{2}\left(t+1\right),s_{3}\left(t+1\right)\right) \end{array}$$

The update $\vec{x}\to \vec{y}$ is performed simultaneously for all neurons.

### 2.2. Statistical Properties

From the 3 × 3 weight matrix *W* = (*w*_*ij*_) of a given three-neuron motif, we first compute the global state **transition probabilities** $p\left(\vec{y}|\vec{x}\right)$ from each possible state $\vec{x}\in {\left\{0,1\right\}}^{3}$ to each possible state $\vec{y}\in {\left\{0,1\right\}}^{3}$. The resulting 8 × 8 matrix can be interpreted as the transition matrix of a Markov process. We also determine the **state probabilities** $p\left(\vec{x}\right)$ for all possible 8 global network states in the stationary equilibrium situation, which are the solutions of the equation

(6) $$\begin{array}{r} p\left(\vec{x}\right)=\underset{\vec{s}}{\sum }p\left(\vec{y}|\vec{s}\right)p\left(\vec{s}\right). \end{array}$$

Finally, we compute the **state pair probabilities**

(7) $$\begin{array}{r} p\left(\vec{x},\vec{y}\right)=p\left(\vec{y}|\vec{x}\right)p\left(\vec{x}\right), \end{array}$$

which completely describe the statistical (dynamical) properties of the three-node motif.

In the case of undisturbed motifs (i.e., with zero noise input), all statistical properties can be computed analytically. For example, the transition probability from the global state $\vec{x}=\left(0,1,0\right)$ to the new global state $\vec{y}=\left(0,1,1\right)$ would be given by

(8) $$\begin{array}{r} p\left(\left(0,1,1\right)|\left(0,1,0\right)\right)=\left[1-\sigma \left(w_{11}\cdot 0+w_{12}\cdot 1+w_{13}\cdot 0\right)\right]\\ \;\cdot \;\left[\sigma \left(w_{21}\cdot 0+w_{22}\cdot 1+w_{23}\cdot 0\right)\right]\\ \;\cdot \;\left[\sigma \left(w_{31}\cdot 0+w_{32}\cdot 1+w_{33}\cdot 0\right)\right]. \end{array}$$

With added noise, however, we have determined these quantities numerically by analyzing long (10^6^ time steps) simulated time series of motif states, starting each run with the global state $\vec{x}\left(t=0\right)=\left(0,0,0\right)$.

### 2.3. Information Theoretic Properties

Based on the above statistical properties of a given motif, we obtain the **state entropy**

(9) $$\begin{array}{r} H\left(X\right)=-\underset{\vec{x}\in {\left\{0,1\right\}}^{3}}{\sum }p\left(\vec{x}\right)\log_{2}p\left(\vec{x}\right), \end{array}$$

where $\vec{x}$ runs through the eight possible states (0, 0, 0), (0, 0, 1), (0, 1, 0), (0, 1, 1), …(1, 1, 1). The state entropy measures the average amount of information circulating in the motif and is ranging from 0 to 3 bit in our case. Since this quantity does not depend on the time step, we have *H*(*X*) = *H*(*Y*).

Furthermore, we obtain the **mutual information** (MI) between successive states

(10) $$\begin{array}{r} I\left(X;Y\right)=\underset{\vec{x}\in {\left\{0,1\right\}}^{3}}{\sum }\underset{\vec{y}\in {\left\{0,1\right\}}^{3}}{\sum }p\left(\vec{x},\vec{y}\right)\log_{2}\left(\frac{p\left(\vec{x},\vec{y}\right)}{p\left(\vec{x}\right)p\left(\vec{y}\right)}\right), \end{array}$$

which can be interpreted as the “**information flux**” within the network.

Maximizing the MI requires (1) that the amount of information circulating in the recurrent network is large, and (2) that information is transmitted with only small loss from one time step to the next. This becomes apparent in the equation

(11) $$\begin{array}{r} I\left(X;Y\right)=H\left(Y\right)-H\left(Y|X\right)\\ \;=H\left(X\right)-H\left(Y|X\right), \end{array}$$

where the “**stochastic information loss**” *H*(*Y*|*X*) measures the variety of output states that can follow after a given input state. In a purely deterministic system, one would have *H*(*Y*|*X*) = 0. However, due to the probabilistic Boltzmann neurons and the resulting stochastic nature of the state-to-state transitions, we expect *H*(*Y*|*X*) > 0. Equation (11) thus states that the MI is the state entropy minus the stochastic information loss. Both quantities on the right side of this equation can be affected, to different degrees, by the presence of noise.

### 2.4. Added Noise

To simulate the effect of external noise on the information flux, continuous random values η_*j*_(*t*) are added to the internal states of the neurons *j* = 1…3 in every time step:

(12) $$\begin{array}{r} z_{j}\left(t\right)\to z_{j}\left(t\right)+\eta_{j}\left(t\right). \end{array}$$

All η_*j*_(*t*) are drawn, independently, from a Gaussian distribution with zero mean and a prescribed standard deviation, here called the **noise level**. The randomly distorted input states then enter Equation (3) as before.

## 3. Results

**Figure 1:**
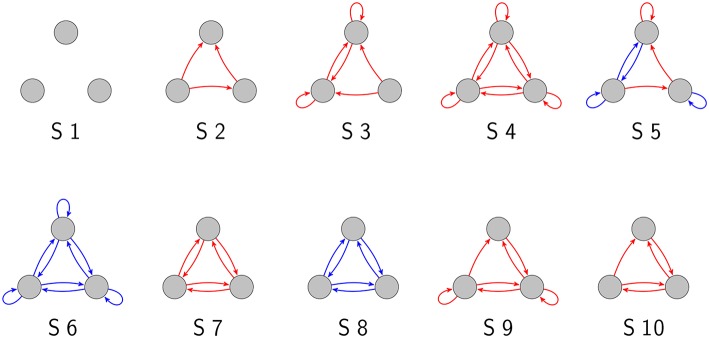
Examples of three-neuron motifs with ternary connections. Gray circles depict neurons, red arrows are excitatory connections (*w*_*ij*_ = 1), and blue arrows are inhibitory connections (*w*_*ij*_ = −1).

As we have demonstrated in a previous publication (Krauss et al., 2019), there are 3,411 topologically distinct three-neuron motifs with ternary connection strengths and possible self-connections (also called “autapses”; Van Der Loos and Glaser, 1972; Bacci et al., 2003; Bekkers, 2009; Yilmaz et al., 2016a,b), ten of which are shown in Figure 1. In the present work, we have investigated these motifs exhaustively with respect to their response to noise.

### 3.1. Noise Response of Specific Motifs

**Figure 2:**
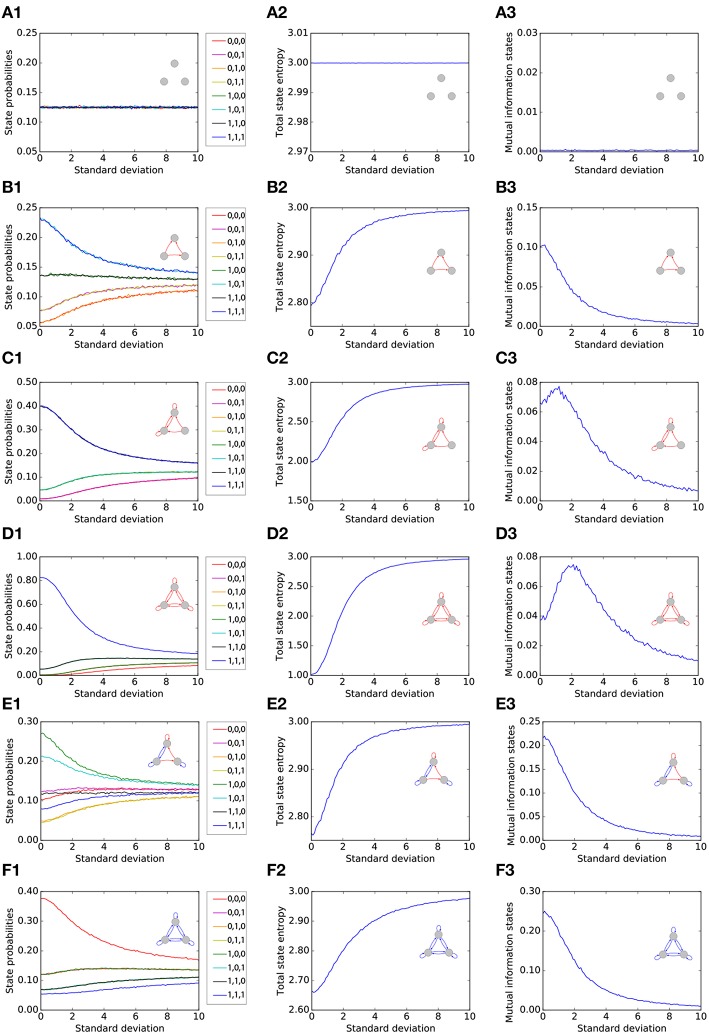
Statistical and information theoretical properties of representative motifs S1 to S6 **(A–F)**, as functions of the noise level. State probabilities $p\left(\vec{x}\right)$ are shown in the left column **(A1–F1)**. State entropy *H*(*X*) is shown in the middle column **(A2–F2)**. Mutual information (MI) of successive states *I*(*X*; *Y*) is shown in the right column **(A3–F3)**. All three quantities are averaged over 10^6^ time steps for each motif and each noise level.

We first single out six representative motifs, two of which show a pronounced effect of Recurrence Resonance. For each of the six motifs (rows of Figure 2), we investigate the statistical and information theoretical properties as functions of the noise level. In particular, we compute the state probabilities $p\left(\vec{x}\right)$ (left column of Figure 2). Furthermore, we compute the state entropy *H*(*X*) (middle column), and the mutual information (MI) of successive states *I*(*X*; *Y*) (right column). All three quantities are averaged over 10^6^ time steps for each motif and each noise level. At the beginning of each run, all three neurons *i* were set to the state *s*_*i*_(*t* = 0) = 0.

#### 3.1.1. Motif S1

We first consider, as a reference, the totally unconnected motif S1 (Figures 2A1–A3). We obtain the expected results: all global states are equally probable with $p\left(\vec{x}\right)=\frac{1}{8}\forall \vec{x}$, and the state entropy is maximal with *H*(*X*) = 3*bit*. However, successive states in this motif are statistically independent and thus have no information in common, so that *I*(*X*; *Y*) = 0*bit*. Furthermore, all quantities are also independent from the noise level, since the system is completely random from the beginning. Due to this noise-independence, motif S1 can of course not exhibit the Recurrence Resonance effect.

#### 3.1.2. Motif S2

We next consider the motif S2 (Figures 2B1–B3), which has three excitatory connections. Without added noise, we find that certain network states, in particular states (1, 0, 1) and (1, 1, 1), are more probable than others, leading to a sub-optimal state entropy of *H*(*X*) ≈ 2.8*bit*. Due to the presence of connections between the neurons, the next motif state is now to some extent predictable from the former one, so that *I*(*X*; *Y*) ≈ 0.1*bit*. As the noise level is increased, all state probabilities start to asymptotically approach the uniform value of $\frac{1}{8}$. Consequently, we find a monotonous increase of the state entropy toward the maximum value of 3 bit, which in principle should favor the information flux in the motif and thus should help to increase the MI between successive states. However, the added noise also increases the stochastic information loss *H*(*Y*|*X*) (not shown in the figure), which has a detrimental effect on the information flux. In this case of motif S2, *H*(*Y*|*X*) is growing faster with the noise level than *H*(*X*). Since *I*(*X*; *Y*) = *H*(*X*) − *H*(*Y*|*X*), this leads to a *monotonous decrease* of the MI from initially 0.1*bit* without noise to almost zero at very large noise levels.

#### 3.1.3. Motif S3

The motif S3 (Figures 2C1–C3) Has already six excitatory connections. The state probabilities and the state entropy show a behavior that is qualitatively similar to motif S2. Quantitatively, however, the state entropy without added noise is now with *H*(*X*) ≈ 2 even less optimal than in motif S2: The system is pinned most of the time in only a few dominating states. As the noise level is gradually increased, the system is freed from these dominating states, and the state entropy *H*(*X*) is now growing faster than the stochastic information loss *H*(*Y*|*X*). Since *I*(*X*; *Y*) = *H*(*X*) − *H*(*Y*|*X*) in a recurrent network, the MI is now initially *increasing* with the noise level, then reaches a maximum at a noise level of about 1 (= standard deviation of Gaussian distribution), and eventually falls to zero for even larger noise levels.

#### 3.1.4. Motif S4

The motif S4 (Figures 2D1–D3) Has the largest possible number of nine excitatory connections. Here, we find an even more pronounced maximum of the MI as a function of the noise level.

#### 3.1.5. Motif S5

So far, all considered motifs contained only excitatory connections. We next investigate motif S5 (Figures 2E1–E3), which has three excitatory but also four inhibitory connections. We find that the presence of inhibition counteracts the effect of Recurrence Resonance. Indeed, the behavior of motif S5 resembles that of motif S2.

#### 3.1.6. Motif S6

Finally, in motif S6, which has the largest possible number of nine inhibitory connections (Figures 2F1–F3), the total state entropy without added noise is lower than in motif S5, but nevertheless we find no Recurrence Resonance effect.

### 3.2. Strength of Recurrence Resonance Among all Ternary Three-Node Motifs

The representative examples above suggest that only a subset of the three-node motifs with ternary connection strength show a pronounced Recurrence Resonance effect, in particular those with many excitatory and few inhibitory connections. In order to test this hypothesis, we have investigated the noise response of all 3,411 motifs individually, by calculating the mutual information of successive states *MI* = *I*(*X*; *Y*) as a function of the noise level σ_*noise*_. We then compared how strong the Recurrence Resonance effect was pronounced in each of the motifs. The strength Δ*MI* was quantified by the relative change of the MI between the zero noise level σ_*noise*_ = 0 and the noise level σ_*noise*_ = σ_*opt*_ where the MI is maximal, that is

(13) $$\begin{array}{r} \Delta MI=\frac{MI\left(\sigma_{noise}=\sigma_{opt}\right)-MI\left(\sigma_{noise}=0\right)}{MI\left(\sigma_{noise}=0\right)}. \end{array}$$

Note that the completely unconnected motif (S1 in Figure 1) had to be taken out of this evaluation, because in this special case *MI*(σ_*noise*_ = 0) = 0.

**Figure 3:**
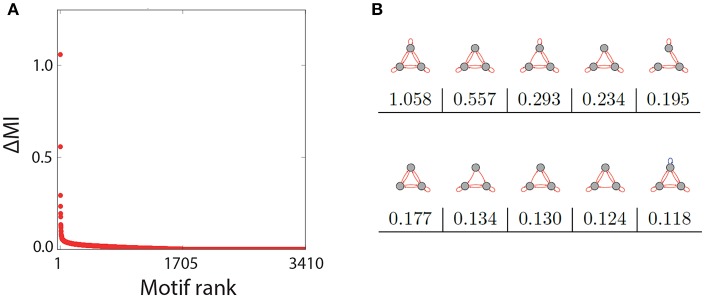
Rank order of motifs with respect to the Recurrence Resonance effect. **(A)** Relative change Δ*MI* of the mutual information *I*(*X*; *Y*) between the optimum noise level and zero noise, as a function of the motif rank. **(B)** The top ten motifs that show the strongest Recurrence Resonance effect, as well as their Δ*MI* values.

After computing Δ*MI* for all remaining 3410 motifs, we have plotted the results in a rank-ordered way (Figure 3A). We find that Δ*MI* ≈ 0 for almost all motifs. Only very few motifs show a significant Recurrence Resonance effect, and the ten top ranking motifs are depicted in Figure 3B, together with their respective Δ*MI* values. The strongest effect is indeed found in the motif that has nine excitatory connections. Interestingly, among the ten top ranking motifs, only the last one has a single inhibitory connection.

### 3.3. Correlation of Recurrence Resonance With Structural Motif Properties

Taken together, the above investigations suggest that the Recurrence Resonance effect occurs preferentially in motifs with many excitatory and few inhibitory connections. To test this hypothesis, and to identify possible other structural motif parameters that might play a role in this context, we have again investigated the set of 3,411 motifs and computed the Spearman's correlation coefficient *r* between Δ*MI* and various other parameters. It turns out that the correlation of Δ*MI* with any of these individual parameters is relatively weak, but in some cases highly significant:

As expected, there is a significant positive correlation (*r* = 0.08, *p* = 8.5 · 10^−6^) between Δ*MI* and the total number of excitatory connections in a motif. By contrast, the correlation with the total number of inhibitory connections is negative (*r* = −0.11, *p* = 2.3 · 10^−11^). Consistently with these observations, there is a positive correlation (*r* = 0.11, *p* = 5.7 · 10^−10^) with the sum $\underset{i,j}{\sum }w_{i,j}$ of all nine weights in a motif. This quantity is similar to a previously defined network weight statistics parameter called “balance” which has been identified to have the largest impact on the dynamical behavior of recurrent neural networks (Krauss et al., 2019).

While each single neuron in our model motifs can have mixed connections of both signs, according to *Dale's principle*, the outgoing connections of biological neurons are either purely excitatory or purely inhibitory (Dale and Gaddum, 1930; Eccles et al., 1954; Strata and Harvey, 1999). Therefore, we have tested if Δ*MI* is affected by the number of neurons in a motif that are “synaptically specialized” in that way. We find however no significant correlation of Δ*MI* with the number of neurons that are specialized on the output side (*r* = 0.03, *p* = 0.05).

Surprisingly, the strongest of all investigated correlations is found between Δ*MI* and the number of motif neurons that are specialized on the input side, i.e., they receive either only excitatory or only inhibitory connections (*r* = 0.12, *p* = 1.5 · 10^−11^).

Finally, we have also investigated how the number of self-connections (autapses) in a motif affects Δ*MI*, but found no significant correlation (*r* = 0.01, *p* = 0.05).

## 4. Discussion

We have investigated in this work how noise affects the “information flux” in recurrent neural networks (RNNs). To facilitate an exhaustive study, we focused strictly on a very simple class of RNNs, namely the 3,411 possible three-node motifs with ternary connection strength. In general, motifs (i.e., frequently recurring wiring patterns) can be viewed as the basic building blocks of various types of networks in nature. In particular, neural motifs can serve useful functions, such as the acceleration and delay of response in long- and short-term memory (Li, 2008).

In our present motif model, we have accounted for the possibility of self-connections from one neuron to itself. Such self-connections, also called *autapses* (Van Der Loos and Glaser, 1972), are excluded from some theoretical studies, but are actually quite common in the nervous system [e.g., 80 percent of all layer 5 pyramidal neurons in rat neocortex build autaptic connections (Lübke et al., 1996)]. Moreover, it has been shown that autapses provide a previously unknown and powerful form of inhibitory synaptic feedback in a particular class of cortical interneurons (Bacci et al., 2003). Autapses also contribute to a positive-feedback loop that maintains persistent electrical activity in neurons (Bekkers, 2009). Even more relevant to the present study, it has been demonstrated that (electrical and chemical) autapses affect the temporal coherence or firing regularity of single stochastic Hodgkin-Huxley neurons and scale-free neuronal networks (Yilmaz et al., 2016b). Interestingly, this study has found that multiple coherence resonance can be induced by a proper choice of the autaptic time delay. Finally, for a specific range of the coupling strength, autapses can significantly improve the propagation of spiking activity from pacemaker neurons (Yilmaz et al., 2016a).

In the present study, we have investigated how certain probabilistic and information-theoretic properties *f* of motifs depend on the level σ_*noise*_ of added noise, and a vast amount of similar *f*(σ_*noise*_)-studies have been conducted in the fields of Stochastic Resoance (SR) and Coherence Resonance (CR).

The phenomenon of SR is typically discussed in the context of signal detectors, or sensors, with a threshold that transmit physical signals from the environment into an information processing system (Benzi et al., 1981; Wiesenfeld and Moss, 1995; Gammaitoni et al., 1998; Moss et al., 2004; Krauss et al., 2016, 2017, 2018). Virtually all sensors have a detection threshold, that is, a minimum required signal intensity below which detection is normally not possible. However, when noise is added to the signal before entering the sensor, even very weak sub-threshold signals can be enhanced above the threshold. From a theoretical point of view, the ideal way to quantify this effect is by computing the MI between the signal input and the sensor output, as this quantity measures the true information transmission across the sensor. If a plot of the MI as a function of the noise level shows a peak, this is considered as the hallmark of SR.

The phenomenon of CR was first demonstrated with an excitable FitzHugh-Nagumo system under external noisy driving (Pikovsky and Kurths, 1997), where it was found that the coherence of the noise-induced oscillations become maximal for a certain noise level. A related effect was later found in the non-linear response of the Hodgkin-Huxley neuron model (Lee et al., 1998).

Researchers have not only investigated the effect of noise on individual neurons, but also on small neuron motifs. A particularly well-studied example is the feed-forward-loop motif, in which neuron 1 drives neuron 2, and neurons 1 and 2 both drive neuron 3. A first study demonstrated that these motifs can exhibit both SR and CR, but that the coupling strength serves as a control parameter for the stochastic dynamics (Guo and Li, 2009). Further work has investigated the effects of time delay on the SR in feed-forward-loop motifs and it was found, among other interesting results, that the correlation between the periodic subthreshold signal's frequency and the dynamical response of the network motifs is resonantly dependent on the intensity of additive spatiotemporal noise (Liu et al., 2014). Finally, researchers studied how SR in feed-forward-loop motifs is affected by astrocytes, which are active partners of neuronal signal processing in the brain. They found that, in the presence of astrocytes, the performance of the motifs on weak signal transmission in both noisy and noise-free environments can be significantly improved (Liu and Li, 2013).

In the present work, we have studied how noise affects the flux of information in motifs of three probabilistic neurons, mutually connected with links of ternary weights. In order to quantify the ongoing information flux in a motif, we used *I*(*X*; *Y*), the MI between successive states. In addition, we considered the state entropy *H*(*X*), which measures the average information content circulating in the network, regardless of whether this information is random or temporally correlated. Finally, we considered the stochastic information loss *H*(*Y*|*X*), which is due to the probabilistic nature of the neurons and due to the added noise.

As was to be expected, added noise increases the state entropy *H*(*X*) by broadening the distribution of system states within the available state space. At the same time, the noise makes state-to-state transitions more random compared to the undisturbed system, and thus also increases the stochastic information loss *H*(*Y*|*X*). However, since a large information flux requires simultaneously a large state entropy and a small stochastic information loss, the effect of noise depends on the relative rates of increase of *H*(*X*) and *H*(*Y*|*X*).

If an undisturbed neural network is already operating in a dynamically rich regime, where all possible system states are visited with approximately the same probability, *H*(*X*) cannot be substantially increased by adding noise. On the other hand, the added noise can lead to a rapid increase of the stochastic information loss *H*(*Y*|*X*), in particular if the undisturbed system is behaving in a relatively deterministic way. As a consequence, such neural networks will show a monotonous decrease of *I*(*X*; *Y*) with the noise level. Indeed, we have observed this type of behavior in weakly connected motifs and in motifs with predominantly inhibitory connections.

A qualitatively different behavior is found in neural networks which are originally “trapped” in a restricted region of state space. In this case, adding just a relatively small amount of noise can quickly “free” the system from its dynamical trap, leading to a rapid increase of the state entropy *H*(*X*). If *H*(*X*) is initially growing faster with the noise level than *H*(*Y*|*X*), this will lead to a maximum of *I*(*X*; *Y*) for some non-zero noise intensity. We have found this type of behavior particularly in motifs with many excitatory connections, and it is interesting to note that such motifs are over-represented in the connectivity scheme of layer 5 pyramidal neurons in the rat cortex.

Thus, our study shows that noise can have either a detrimental or a beneficial effect on the information flux in recurrent neural networks, and adding noise can be particularly useful in systems that operate within a dynamically sub-optimal regime. Whether this Recurrence Resonance effect is also present in larger neural networks and with different types of neurons remains to be shown in future studies. Nevertheless, we speculate that the brain could use bursts of noise to free neural network dynamics from being permanently trapped in “attractor states.”

## Data Availability

The datasets generated for this study are available on request to the corresponding author.

## Author Contributions

PK and CM designed the study, developed the theoretical approach, and wrote the paper. KP and PK performed computer simulations. PK, CM, KP, and AS discussed the results. PK, KP, and AS prepared the figures. All authors read and approved the final manuscript.

### Conflict of Interest Statement

The authors declare that the research was conducted in the absence of any commercial or financial relationships that could be construed as a potential conflict of interest.
